# Breaking the Freeze: The Role of Cognitive Function in Freezing of Gait in Parkinson’s Disease

**DOI:** 10.1155/padi/7199074

**Published:** 2026-07-21

**Authors:** Ondrej Papacek, Evzen Ruzicka

**Affiliations:** ^1^ Department of Neurology, Center of Clinical Neuroscience, First Faculty of Medicine, Charles University, Prague, Czech Republic, cuni.cz; ^2^ Department of Neurology, General University Hospital in Prague, Prague, Czech Republic

**Keywords:** cognitive training, dual-task training, executive dysfunction, freezing of gait (FOG), Parkinson’s disease (PD)

## Abstract

Freezing of gait (FOG) is a highly disabling, poorly dopa‐responsive symptom of Parkinson’s disease (PD) that becomes increasingly prevalent with disease progression and is one of the main contributors to falls and loss of independence. Although FOG has long been viewed as a motor phenomenon, converging evidence shows that executive impairment—particularly deficits in attention, task‐switching, inhibition, and visuospatial processing—is strongly implicated in its pathophysiology. Yet the field lacks a coherent framework explaining how these cognitive processes contribute to FOG and how they should be targeted in therapy. This narrative review synthesizes current evidence on the neural mechanisms linking cognitive dysfunction and FOG. Literature was identified through targeted searches of neuroimaging, behavioral, and rehabilitation studies in PD with and without FOG. Findings consistently demonstrate altered activity and connectivity within corticostriatal and corticolimbic circuits in freezers, including inefficient hyperactivation of the frontal, prefrontal, and posterior parietal cortices, and abnormal coupling between the ventral striatum, precuneus, and amygdala. These patterns suggest that cognitive networks become overrecruited yet insufficient to compensate for impaired motor automaticity, especially under dual‐task demands or emotional load. Robust cognitive reserve can serve as compensation, whereas cognitive impairment can contribute to FOG in situations with high cognitive load. Freezers show disproportionate deficits in inhibitory control, visuospatial processing, and task‐switching, which correlate with gait initiation failures and FOG severity. Cognitive training, particularly dual‐task and other motor–cognitive interventions, shows promising yet variable effects on gait, executive performance, and FOG; however, the field lacks clarity about which cognitive and executive domains are causally involved and which merely reflect compensatory strain. FOG‐specific mechanistic models integrating motor, executive, and limbic dysfunction are needed to guide individualized cognitive training and optimize therapeutic outcomes for people with PD.

**Summary**
• Plain Language Title
◦ Can training the brain help stop freezing of gait?
• Plain Language Summary
◦ FOG is a common and disabling symptom in people with PD. It refers to brief moments when a person suddenly cannot move their feet forward, especially during turning, walking through doorways, or multitasking. Although FOG looks like a purely movement problem, research shows it is closely linked to cognitive difficulties, especially with executive functions. Executive functions are high‐level brain skills such as paying attention, switching between tasks, controlling impulses, and processing visual information. Brain scans of people with Parkinson’s who experience FOG show changes in areas of the brain responsible for planning, attention, and decision‐making. These individuals often show more activity in the prefrontal and parietal parts of the brain, possibly because they need to work harder to move. They also show abnormal connections between brain regions that control emotion and movement, which may explain why stress or anxiety can trigger FOG. Training programs that target executive functions, especially using dual‐task exercises where people do a mental and physical task at the same time, have shown promising results in reducing FOG. These interventions may help by strengthening the brain’s ability to compensate for weaknesses. However, scientists still do not fully understand if executive dysfunction causes FOG or if it develops as a result of it. There are also unanswered questions about what type of training is best. Should it be purely mental, purely physical, or a combination of both? Should training be general or tailored to each person’s specific cognitive weaknesses? This review highlights the strong link between FOG and thinking problems in Parkinson’s and emphasizes the need for more targeted, personalized therapies. A better understanding of the brain changes involved could lead to improved treatments that help people with Parkinson’s walk more safely and confidently.

## 1. Introduction

Parkinson’s disease (PD) is one of the most common neurodegenerative disorders, and its prevalence is expected to double in the next 20 years [1]. While dopamine replacement therapy improves motor symptoms such as bradykinesia and rigidity, less dopamine‐responsive symptoms such as freezing of gait (FOG) emerge as the disease progresses [2]. A FOG episode is defined as a period of paroxysmal inability to take an effective step toward the intended direction of attempted travel, which does not resemble the typical or quasinormal stepping performance exhibited by the same individual during similar gait tasks or comparable conditions [3]. FOG typically manifests during gait initiation or cessation, turning, or when passing through a doorway [4] and affects more than 30% of patients in early PD and up to 80% in later disease stages [2, 5]. It represents one of the most important risk factors for falls in PD and a major contributor to reduced quality of life in affected individuals [6]. Notably, FOG is associated with a distinct profile of cognitive and executive dysfunction that remains significant even when accounting for disease duration and overall severity [7, 8]. Executive or motor–cognitive training approaches, including dual‐task paradigms, have shown promising effects on FOG, although substantial questions remain regarding their optimal application and mechanisms of action [9–12]. The extent to which cognitive dysfunction contributes to FOG—whether as a causal factor, a consequence, or a compensatory mechanism—remains unclear. Therefore, the aim of this review is to provide a narrative framework linking FOG pathophysiology, cognitive dysfunction, and interventions based on motor–cognitive training.

## 2. Clinical Manifestation of FOG

FOG presents in several phenomenological variants, including akinetic, kinetic‐trembling, and kinetic‐no‐trembling. A single FOG episode may involve only one or all of these manifestations [3]. Kinetic‐trembling FOG, the most common form, involves fast oscillatory movements in the lower limbs and a sensation of being “stuck” [13]. Kinetic‐no‐trembling FOG is characterized by any other ineffective movement visible in the legs and feet that is not clearly trembling, including slow irregular oscillatory movements, paroxysmal shuffling, and festinating‐freezing with a forward‐shifted center of gravity [3]. Akinetic FOG manifests as an inability to initiate or sustain walking with no clinically observable movements in the lower limbs [14, 15]. A FOG onset is the first foot lift during an ineffective step or the first observable, self‐reported, or documented attempt to initiate movement. FOG termination refers to the first foot lift, marking a return to typical stepping (two consecutive normal steps) or the cessation of attempts, including falls [3].

## 3. Subtypes of FOG

FOG is a highly heterogeneous phenomenon, with several theoretical subtypes—including asymmetric‐motor, anxiety‐related, and sensory‐attentional forms—proposed to categorize common triggers [16, 17]. These proposed subtypes are increasingly regarded as conceptual frameworks rather than distinct phenotypes, as methodological limitations, small sample sizes, and the inherent heterogeneity of PD necessitate caution against rigid classification [18, 19]. This individual variability likely extends to FOG itself [16, 17]. The clinical expression of FOG is significantly modulated by dopaminergic medication, manifesting as either “OFF‐FOG” (occurring when medication is suboptimal) or “ON‐FOG” (occurring despite adequate dopaminergic therapy) [11]. Notably, up to 38% of patients experience ON‐FOG [20], a phenomenon that underscores the critical involvement of nondopaminergic systems, such as the cholinergic and noradrenergic pathways, in the pathophysiology of freezing [21–24]. Despite the diverse clinical presentations and medication states, common cognitive vulnerabilities appear to link these various triggers and subtypes [7, 8]. This dependency on attentional and executive resources for compensation, necessitated by the loss of gait automaticity, appears to be a common feature shared across all clinical triggers and subtypes [25–27].

### 3.1. Cross‐Talk Model Hypothesis

The cross‐talk model proposes that FOG arises from excessive interaction between normally segregated basal ganglia circuits, resulting in information overload within the striatum and overstimulation of basal ganglia output nuclei [28, 29]. Specifically, the traditionally distinct motor, cognitive, and limbic circuits become increasingly integrated, allowing nonmotor signals to interfere with gait control [29, 30]. The first functional neuroimaging evidence supporting this model was provided by Shine et al. (2013), who demonstrated increased frontoparietal cortical activation during FOG, suggesting aberrant engagement of cognitive control networks during gait [31]. Subsequent studies have further supported the cross‐talk hypothesis, showing that greater coupling of the ventral putamen with limbic (amygdala) and cortical (precuneus) regions is associated with increased FOG severity [32, 33]. Conversely, reduced coupling—or “decoupling”—between these networks is linked to successful compensation and FOG resolution [32]. These changes are often accompanied by hypoconnectivity between the putamen and frontoparietal executive networks, a pattern associated with heightened anxiety and more severe freezing [34].

Together, these alterations suggest that excessive limbic and cognitive influence on basal ganglia motor circuits overloads striatal processing, propagates to basal ganglia output nuclei, and ultimately disrupts locomotor control at the level of brainstem gait centers [17, 29, 30]. Interestingly, the increased integration of attentive and cue‐response networks plays a dual role: while it can trigger FOG under high cognitive demands, it also serves as a critical compensatory mechanism [16, 17]. This compensation is likely mediated by the increased recruitment of frontal, prefrontal, and parietal cortices, as well as cerebellar networks, which help regulate activity and maintain gait stability [25, 26, 35].

### 3.2. Cognitive Dysfunction in FOG

Cognitive impairment is common in people with PD who experience FOG, and its presence early in the disease course is associated with an increased risk of developing FOG [29, 36]. Cognitive impairment and FOG frequently co‐occur, with their prevalence increasing with disease severity and duration, reaching up to 80% in advanced stages [2, 7, 37, 38]. Crucially, FOG is significantly linked to impairments in global cognition, attention, visuospatial processing, and executive functions—particularly response inhibition and task‐switching—even after controlling for disease duration and severity [7, 8]. Nonetheless, these findings suggest that cognitive dysfunction represents a significant contributing factor rather than the primary or universal determinant of FOG, highlighting the multifactorial nature of the phenomenon [7, 16, 39].

### 3.3. Neurobiological and Neurochemical Foundations

Cognitive dysfunction in FOG is deeply rooted in the impairment of multiple neurotransmitter systems beyond the dopaminergic pathways, providing a mechanistic explanation for the emergence of ON‐FOG [22, 23]. Specifically, impairments in cholinergic signaling are further associated with overall cognitive decline, reduced gait speed, and poor performance during gait dual‐tasking [22, 23]. In addition, the noradrenergic system influences FOG through its modulatory role in arousal [28]. This level of arousal and emotional state also impacts executive functions, which in turn significantly influence FOG susceptibility [40].

In support of the cross‐talk model, neuroimaging studies indicate that nonfreezers can partially compensate for impaired sensorimotor striatal function by recruiting frontal, parietal, and cerebellar networks to maintain gait stability [25, 26, 35]. In contrast, freezers show reduced capacity to effectively recruit cognitive frontal regions required to maintain motor output while responding to task demands or environmental cues [25, 41, 42]. Altered connectivity between the ventral striatum and cortical–limbic networks is consistently observed in freezers and correlates with worse dual‐task performance, supporting the notion that impaired integration of executive and motor control contributes to FOG vulnerability [32–34]. Freezers also demonstrate increased activation in the dorsolateral prefrontal cortex (dlPFC) and posterior parietal cortex—core nodes of the frontoparietal executive network supporting attention, task‐switching, and interference resolution [13, 31]. This heightened activation is likely a reflection of a compensatory effort response to impaired automatic gait control [25, 26, 28]. The inefficiency of the frontoparietal network is reflected by the persistence or worsening of gait disturbances, particularly during cognitively demanding situations [13, 26, 33].

Consistent with these neuroimaging findings, this heightened dlPFC activation correlates at the behavioral level with greater dual‐task cost, impaired gait kinematics, and abnormal anticipatory postural adjustments, particularly during high‐risk tasks such as turning [13]. In parallel, freezers frequently exhibit deficits in inhibitory control, affecting both gait initiation and stopping [43, 44]. Problems with initiating and stopping gait also respond poorly to cueing [15]. One prevailing explanation suggests a failure to appropriately link the gait cycle with step generation [24, 45, 46]. Furthermore, worse performance during dual‐tasking is associated with increased activity in the inferior frontal gyrus and the supporting motor area (SMA), both of which are involved in movement initiation [47]. At the same time, there is decreased activation in the primary motor cortex, caudate, and supramarginal areas. The supramarginal gyrus, much like the precuneus, is related to visuospatial processing [47]. However, findings are not fully consistent across studies: while several reports demonstrate impaired response inhibition in freezers using go/no‐go paradigms [43, 44], others offer contrary evidence [39]. Other studies suggest preserved inhibitory circuitry, implying that inhibitory deficits may reflect overload or inefficiency of prefrontal control rather than a primary failure of inhibitory motor networks per se [44, 48].

### 3.4. Compensatory Mechanisms and Network Interference

Compensatory mechanisms therefore appear to differ between nonfreezers and freezers. In nonfreezers, increased frontoparietal activity effectively compensates for sensorimotor striatal dysfunction, whereas in freezers, this executive compensation appears to fail and is replaced by hyperconnectivity of the precuneus and ventral striatum [33]. This altered pattern is associated with increased cognitive load and poorer dual‐task performance, suggesting an inefficient or maladaptive compensatory strategy rather than effective executive control [33]. According to the cross‐talk hypothesis, FOG may be exacerbated due to the integration of motor and cognitive networks [11, 17, 30]. Increased coupling between the cognitive and the limbic or motor networks is one of the key mechanisms of cognitive interference in FOG [32]. On the other hand, low capacity of cognitive networks means limited compensation possibilities [25, 26]. The role of cognitive function in FOG represents a double‐edged sword. While preserved executive capacity and increased activation in prefrontal, frontal, and parietal regions are crucial for overcoming FOG—particularly during cueing—this reliance can also exacerbate freezing when heightened circuit cross‐talk overwhelms limited resources [10, 13, 25, 26, 32, 35]. Consequently, freezers may rely more heavily on cognitive control than nonfreezers due to a greater loss of movement automaticity [25, 26, 41, 42]. This inherent heterogeneity likely explains inconsistencies across studies and underscores the importance of individualized models of FOG pathophysiology [16, 17]. A summary of the changes in connectivity and function of neural circuits important for FOG is shown in Table 1.

**TABLE 1:** Cognitive/neural mechanisms and their functional significance for FOG.

Cognitive/neural mechanism	Study	Changes in connectivity/function	Functional correlate relevant to FOG
Dual‐task interference and attentional load	Vervoort et al., [33]	Hyperconnectivity putamen–precuneus Hypoconnectivity ventral striatum–superior temporal lobe	Worse dual‐task performance, longer double support times in gait, FOG present
	Sarasso et al. [47]	Increased activity in inferior frontal gyrus and supplementary motor area; decreased activity in primary motor cortex, caudate nucleus, and supramarginal gyrus	Worse dual‐task performance, turning, and executive function
	Moreira‐Neto et al. [13]	Increased activation of dlPFC and posterior parietal cortex Decreased activation of MLR	Worse dual‐task performance, more severe FOG during turning, smaller APAs during gait initiation

Executive control (initiation and inhibition)	Moreira‐Neto et al. [13]	Increased activation of dlPFC and posterior parietal cortex Decreased activation of MLR	Worse FOG, worse gait initiation
	Sarasso et al. [47]	Increased activity in inferior frontal gyrus and supplementary motor area; decreased activity in primary motor cortex, caudate nucleus, and supramarginal gyrus	Worse turning and executive function

Frontoparietal executive network recruitment	Shine et al. [31]	Increased activity in frontoparietal cortical regions Decreased activity in globus pallidus internus, caudate nucleus, and MLR	Greater FOG severity severity during the TUG test
	Shine et al. [41]	Decreased activity in the cognitive network–anterior insula, ventral striatum, and the presupplementary motor area	Higher FOG‐Q total, more out‐of‐sequence steps and delayed footstep responses
	Shine et al. [42]	Decoupling between the cognitive network and the basal ganglia network	Paroxysmal motor arrests similar to FOG
	Moreira‐Neto et al. [13]	Increased activation of dlPFC and posterior parietal cortex Decreased activation of MLR	Worse dual‐task performance, more severe FOG during turning, smaller APAs during gait initiation

Limbic–executive cross‐talk	Ehgoetz Marten et al., [32]	Coupling between cortical and limbic networks Anticoupling between ventral striatum and cortical/limbic networks	Increased FOG severity Increased FOG compensation
	Gilat et al. [34]	Hyperconnectivity putamen–amygdala De‐coupling putamen–frontoparietal network, decoupling frontal networks–amygdala	Increased FOG severity, worse anxiety, and fear of falling

*Note:* Connectivity and functional alterations of neural circuits implicated in freezing of gait. Some studies are classified into multiple categories of neural mechanisms since they involve multiple components of cognition. dlPFC, dorsolateral prefrontal cortex; APAs, anticipatory postural adjustments; TUG, timed up and go test.

Abbreviations: FOG‐Q, Freezing of Gait Questionnaire; MLR, mesencephalic locomotor region.

## 4. The Interplay of Executive Function and Anxiety in FOG

Anxiety is a well‐established contributor to and predictor of FOG in PD [36]. Although dual‐tasking exacerbates FOG in most patients, its impact appears particularly pronounced in individuals with an anxiety‐related FOG subtype [18]. Through its impact on prefrontal function, anxiety compromises the very executive processes—such as attention allocation and task‐switching—that freezers rely on for gait stability [34]. Consistent with this, freezers report higher anxiety during gait compared to standing, and anxiety‐related FOG shows limited responsiveness to dopaminergic medication [18, 49]. Heightened arousal engages limbic circuitry, particularly the amygdala, biasing behavior toward stimulus‐driven and habitual motor responses at the expense of goal‐directed executive control [34, 40].

**FIGURE 1:**
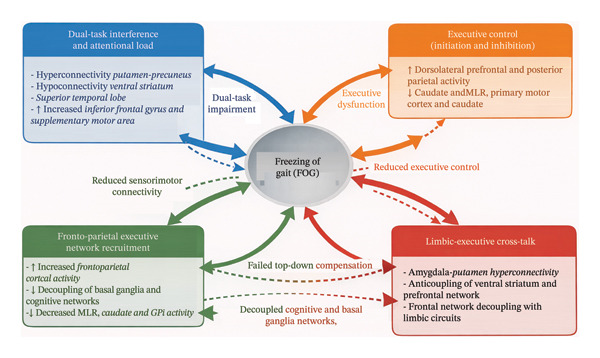
The diagram illustrates network‐level alterations associated with FOG. Increased attentional load and dual‐tasking are linked to heightened engagement of frontoparietal executive regions and premotor areas, alongside reduced activity in primary motor cortex, caudate nucleus, supramarginal gyrus, and the mesencephalic locomotor region (MLR). Impaired executive control of gait initiation and inhibition involves similar cortical–brainstem interactions. Frontoparietal network recruitment reflects compensatory cortical engagement during gait and cueing, accompanied by reduced basal ganglia output and disrupted cognitive–basal ganglia coupling. Limbic–executive cross‐talk, characterized by increased limbic–striatal coupling and reduced frontoparietal regulation, is associated with greater anxiety, fear of falling, and increased FOG severity.

In PD, where habitual motor control is already compromised, this limbic dominance further disrupts movement initiation and adaptation and reinforces the cross‐talk model, where reduced prefrontal regulation allows limbic circuits to interfere with motor output [32, 33, 50]. By diverting attentional resources toward threat monitoring, rumination, or goal‐irrelevant stimuli, anxiety‐driven arousal deprives patients of the conscious executive resources needed to bridge the gap left by compromised gait automaticity [25–27]. Reduced connectivity between frontoparietal executive networks and the amygdala further weakens top‐down emotional regulation, potentially reinforcing maladaptive limbic–motor interactions [34]. Over time, persistent limbic–cortical dysregulation may contribute to caudate hypoconnectivity and gray matter loss, a structural feature reported in freezers [51]. Clinically, these findings suggest that anxiety‐related FOG reflects an interaction between heightened arousal and limited executive control capacity [40]. Interventions targeting cognitive control or emotional regulation, such as mindfulness‐based approaches or structured cognitive training, may therefore support gait by strengthening prefrontal–limbic communication, particularly in patients for whom anxiety is a prominent trigger [52]. The current understanding of how cognitive function relates to FOG is summarized in Figure 1.

## 5. Executive and Cognitive Training Strategies for FOG

Freezers often rely on compensatory strategies based on attentional focus or external cueing to mitigate or overcome FOG [25–27]. These strategies include visual and auditory cues, shifts in attentional focus, weight‐shifting maneuvers, and other context‐specific compensations [15, 53]. When appropriately applied, such strategies can reduce fall risk and effectively alleviate FOG during episodes [53]. FOG‐specific training that emphasizes cueing is therefore clinically useful and is often preferred by freezers over more cognitively demanding executive strategies [15]. However, these approaches primarily address FOG once it occurs, rather than targeting underlying mechanisms that increase susceptibility to freezing. Importantly, cueing strategies themselves require sufficient attentional capacity and intact frontal and parietal network function, limiting their effectiveness in individuals with reduced executive reserve [25, 26].

Another critical aspect of FOG interventions is task specificity, defined as training attentional and cognitive resources in environments and situations where freezing most commonly occurs. These “hotspots,” typically located in patients’ homes, often include narrow spaces such as doorways, corridors, or bathrooms [16]. A systematic review of training‐based FOG interventions identified the so‐called FOG‐correlates category as the most promising approach. This category comprises exercises designed to enhance resilience against freezing, including motor–cognitive tasks such as dual‐task gait training or Stroop‐based paradigms performed during gait. The pooled effect size for FOG‐correlates interventions was 0.40, compared to 0.14 for generic exercise programs [10]. Effective interventions frequently incorporated cognitively demanding motor tasks, such as gait with visual restriction, alongside dexterity and balance training in challenging environments, thereby targeting visuospatial and executive processing demands. Group‐based training was another common feature of successful interventions, likely reflecting increased cognitive and executive load due to social and environmental complexity [54, 55].

### 5.1. Motor–Cognitive Gait Training

Dual‐task gait training appears to enhance executive reserve and improve FOG‐related outcomes more effectively than single‐task training [56]. A meta‐analysis of 14 randomized controlled trials (RCTs) including 548 participants demonstrated significant improvements in gait speed, stride length, cadence, and reductions in dual‐task cost following dual‐task interventions [56, 57]. Although concerns have been raised regarding insufficient training intensity in some control conditions [58], findings from the DUALITY trial suggest that improvements may reflect enhanced cognitive reserve rather than task‐specific learning alone [59]. In this trial, increased gait velocity was observed across both trained and untrained dual‐task conditions, with improvements ranging from 7.75% to 13.44% and persisting at 12‐week follow‐up [59]. Given that freezers often exhibit a gradual deterioration of gait prior to FOG onset, dual‐task training may help maintain gait stability across varying cognitive demands, thereby reducing freezing risk [16, 60].

Two RCTs specifically targeting FOG with dual‐task interventions reported moderate improvements in New FOG Questionnaire (N‐FOG) scores (effect size: 0.42), although objective FOG measures did not show significant change [9, 54]. The validity of the N‐FOG as an outcome measure has been questioned, and evidence for objective reductions in FOG remains limited [61]. Nevertheless, neuroimaging studies indicate that dual‐task training is associated with reduced prefrontal cortex activation alongside increased cerebellar connectivity, suggesting improved neural efficiency and attentional capacity [35, 62]. Notably, interventions aimed at enhancing general executive capacity appear more effective than narrowly focused cueing strategies alone [10]. Optimal FOG interventions may therefore require a combination of context‐specific training and broader cognitive capacity enhancement. Table 2 summarizes the RCTs that examined the effect of motor–cognitive and cognitive training on FOG.

**TABLE 2:** Interventions targeting FOG that include cognitive/cognitive‐motor training.

Study	Type of study	(*n*)	Only FOG patients	Intervention	Outcome measure	Main finding
King et al. [54]	RCT	46	Yes	Dual‐task agility gait training (80 min sessions, 3 ×/week, 6 weeks)	N‐FOG‐*Q*, quantitative FOG ratio	Exercise intervention with cognitive challenges (but not education) improves FOG
Clerici et al. [9]	RCT	52	Yes	Multidisciplinary intensive rehabilitation treatment (MIRT) (3–4 h, 3 ×/week, 4 weeks)	FOG‐Q	MIRT is effective for FOG; no added benefit with aquatic therapy
Walton et al. [64]	RCT	38	Yes	Group cognitive training (2 h sessions, 2 ×/week, 7 weeks)	% Time spent frozen (ON/OFF)	Cognitive training reduces time spent in FOG
Lin et al. [65]	Meta‐analysis	337	No	Meditation of mindfulness‐based relaxation (MSBR)	MDS‐UPDRS III. PDQ‐39	Mindfulness and meditation improve MDS‐UPDRS III and PDQ‐39 but not gait velocity, anxiety, or depression
Johansson et al., [57]	Meta‐analysis	597	No	Motor–cognitive training (traditional or virtual reality)	Dual‐task gait speed, cadence, stride length, and cost on gait speed (%)	Motor–cognitive training improves dual‐task gait parameters compared to controls
Killane et al., [66]	Clinical trial	20	No	Dual motor–cognitive virtual reality training (8 × 20 min sessions, 2 weeks)	Motor performance (stepping time, symmetry, and rhythmicity), Cognitive performance (accuracy and reaction time)	Significant improvement in dual‐task performance (motor and cognitive measures)
Strouwen et al. [60]	RCT	121	No	Integrated dual‐task training (IDT) vs. consecutive task training (CTT) 45 min 2x per week 6 weeks,	Gait velocity during the auditory Stroop task	Both IDT and CTT improved dual‐task gait velocity; no significant difference between groups
Bezerra et al., [67]	RCT	39	Yes	Action observation (AO) + motor imagery (MI) + gait training (1 ×/week, 12 weeks)	FOG‐Q	No significant effect of AO + MI + gait training on FOG severity
Silva‐Batista et al. [25]	RCT	32	Yes	Adapted resistance training with instability and dual‐tasking (80–90 min, 3 ×/week, 12 weeks)	N‐FOG‐*Q*, FOG ratio during the turning task	Resistance training with instability and dual‐tasking is superior to resistance training alone for FOG improvement
Sarasso et al. [56]	Meta‐analysis	548	No	Dual‐task gait training vs. single‐task gait training	Dual‐task gait speed, stride length, cadence, dual‐task cost, and quality of life	Dual‐task training improves dual‐task gait speed, stride length, cadence, and quality of life compared to single‐task training

*Note:* This table describes studies evaluating the effect of cognitive/dual‐task training on FOG. Studies examining the effect of this training on gait parameters that are precursors of FOG, such as reduced walking speed, higher dual‐task cost, increased stride variability, and cadence, are included. PDQ‐39 = Parkinson’s Disease Questionnaire.

Abbreviations: AO = action observation, CTT = consecutive task training, FOG‐Q = Freezing of Gait Questionnaire, IDT = integrated dual‐task training, MDS‐UPDRS III = MDS‐Unified Parkinson’s Disease Rating Scale Part III, MI = motor imagery, MIRT = multidisciplinary intensive rehabilitation treatment, N‐FOG‐Q = New Freezing of Gait Questionnaire.

### 5.2. Cognitive and Mindfulness Training

Cognitive training has demonstrated significant benefits for FOG in selected subgroups of freezers, including reductions in freezing duration during the timed up and go test (Cohen’s *d* = 1.02) [63]. Although the experimental group had worse FOG in the ON state than controls, the control group had more severe FOG in the OFF state, suggesting heterogeneity in FOG subtypes. The experimental group showed minimal medication response, indicating that cognitive dysfunction may have played a more prominent role in their FOG, potentially explaining their greater responsiveness to cognitive training [63].

Mindfulness and meditation‐based cognitive training have shown benefits for motor (UPDRS III) and cognitive function in PD, as demonstrated in a recent meta‐analysis of RCTs [64]. Table 2 includes studies investigating the effect of cognitive/mindfulness‐based training on FOG, motor, and cognitive functions. Although earlier studies reported reductions in anxiety and depression [65, 68], these effects were not confirmed in the meta‐analysis, likely due to the short duration (< 3 months) of most included interventions [64]. Nevertheless, an RCT by Kwok et al. (2019) demonstrated comparable improvements in motor symptoms and mobility, along with additional benefits for psychological distress and well‐being [65]. Long‐term mindfulness practice can induce structural changes in brain regions which are key in the pathophysiology of FOG, namely, the caudate nucleus [69]. This is notable, given that caudate gray matter loss represents one of the most prominent structural abnormalities observed in freezers [51]. Both motor–cognitive, and cognitive/mindfulness interventions share some key features regarding frequency and duration of training. Table 3 shows the average values from the RCTs included in Table 2.

**TABLE 3:** Average values of training variables in motor‐cognitive interventions.

Parameter	Average value across RCTs (range)
Frequency of training	2–3 times per week (2–3)
Total duration of training	6–10 weeks (4–12)
Session duration	90 min (45–120)
Total number of sessions	14–30 sessions (8–36)
Supervised vs. unsupervised	Mostly supervised

Table 3 describes the average values of individual parameters from RCTs included in Table 2. For clarity and simplicity, ranges of values are given instead of arithmetic averages.

## 6. Discussion

The present synthesis indicates that cognitive dysfunction contributes to FOG in PD in a heterogeneous and context‐dependent manner, rather than constituting a universal or primary cause [7, 16, 39]. Cognitive impairment is common among individuals with FOG and is associated with an increased risk of developing freezing, particularly when present early in the disease course [29, 36]. However, the frequent co‐occurrence of cognitive impairment and FOG—both of which increase with disease severity and duration—suggests partially overlapping but nonidentical mechanisms [2, 7, 37, 38].

Neuroimaging studies support a model in which cognitive networks modulate gait control by compensating for impaired sensorimotor striatal function, but only within the limits of available cognitive capacity [13, 25, 26, 35]. Nonfreezers appear able to recruit frontoparietal and cerebellar networks to stabilize gait, whereas freezers show reduced efficiency in engaging frontal executive regions during cognitively demanding gait [25, 31, 41, 42]. This pattern suggests that executive dysfunction in FOG may reflect a failure of compensation rather than a primary executive deficit per se [33]. It is plausible that a robust cognitive capacity allows for decoupling between cognitive networks and the putamen, which is an important mechanism for overcoming FOG [32].

In freezers, increased activation of the dorsolateral prefrontal and posterior parietal cortices likely represents an attempt to exert top‐down control over gait in the context of reduced automaticity [13, 25, 31]. However, the association between heightened executive activation and greater dual‐task cost, impaired gait kinematics, and increased FOG severity indicates that this compensation is often inefficient under high cognitive load [13, 26]. The replacement of effective executive compensation by hyperconnectivity of the precuneus and ventral striatum further supports maladaptive network reorganization in freezers, associated with increased cognitive load and poorer dual‐task performance [33].

These mechanistic insights are clinically relevant in light of emerging evidence from cognitive and motor–cognitive training interventions. Dual‐task gait training and other motor–cognitive approaches have demonstrated moderate improvements in subjective FOG severity and consistent benefits for gait parameters and dual‐task performance, suggesting an enhancement of executive reserve rather than a direct normalization of freezing mechanisms [9, 35, 54, 56, 59]. Neuroimaging findings further indicate that such training may reduce prefrontal overactivation while strengthening cerebellar and distributed attentional networks, consistent with more efficient executive engagement [35, 62]. Cognitive and mindfulness‐based interventions have also shown benefits for FOG severity, motor symptoms, and executive performance [63–65].

Anxiety provides an important modulatory context for these effects, is a robust predictor of FOG, and appears to exacerbate freezing by reducing prefrontal regulatory capacity while enhancing limbic influence over motor output [34, 36, 40]. In PD, where habitual motor control is already compromised, this shift toward limbic‐driven behavior further increases vulnerability to freezing, particularly under dual‐task or high‐arousal conditions [32, 33, 50]. Taken together, these findings support a framework in which cognitive function plays a dual role in FOG: as a compensatory resource that can stabilize gait in some individuals—particularly during cueing or structured training—and as a limiting factor when capacity is insufficient or inefficiently recruited [25, 26, 32]. This heterogeneity likely explains inconsistencies across studies and underscores the need for individualized models of FOG pathophysiology and targeted rehabilitation strategies that consider both executive capacity and affective modulation [7, 11].

## 7. Conclusion

FOG in PD reflects a heterogeneous syndrome arising from the interaction of motor, cognitive, and affective mechanisms rather than a single causal pathway [16, 17]. Evidence reviewed here indicates that cognitive dysfunction is a relevant contributing factor in a substantial subgroup of patients, but it is neither necessary nor sufficient to explain FOG across all individuals [7, 39]. Cognitive and executive networks can serve a compensatory role by supporting gait control when automaticity is impaired, particularly during cueing or structured motor–cognitive tasks; however, limited capacity or inefficient recruitment of these networks may also exacerbate freezing under high cognitive or emotional load [13, 26]. Anxiety further modulates this balance by reducing prefrontal control and increasing limbic influence on motor output, thereby increasing vulnerability to FOG [34, 36, 40]. Interventions targeting cognitive capacity, motor–cognitive integration, and emotional regulation show promise for selected patients, underscoring the need for individualized, mechanism‐informed rehabilitation approaches [10, 11, 65].

## Author Contributions

Ondrej Papacek (first author): conceptualization, methodology, investigation, formal analysis, writing–original draft, visualization, and project administration. Evzen Ruzicka: writing–review and editing and supervision.

## Funding

This study was supported by the project National Institute for Neurological Research (Program EXCELES, ID Project No. LX22NPO5107), funded by the European Union–Next Generation EU; the Czech Health Research Council (Grant no. NW24‐04‐00259); and the Charles University: Cooperatio Program in Neuroscience.

## Ethics Statement

The authors have nothing to report.

## Consent

The authors have nothing to report.

## Conflicts of Interest

The authors declare no conflicts of interest.

## Data Availability

Data sharing is not applicable to this article as no datasets were generated or analyzed during the current study.
